# Soft corals form aragonite-precipitated columnar spiculite in mesophotic reefs

**DOI:** 10.1038/s41598-018-37696-z

**Published:** 2019-02-04

**Authors:** Erez Shoham, Thomas Prohaska, Zahava Barkay, Andreas Zitek, Yehuda Benayahu

**Affiliations:** 1 School of Zoology, Faculty of Life Sciences, Tel Aviv University, Ramat Aviv, Tel Aviv 69978 Israel; 2 University of Natural Resources and Life Sciences, Department of Chemistry - VIRIS Laboratory, Konrad Lorenz Strasse 24, A-3430 Tulln, Austria; 3 Wolfson Applied Materials Research Center, Tel Aviv University, Ramat Aviv, Tel Aviv 69978 Israel

## Abstract

Surveys conducted in Eilat’s upper mesophotic coral ecosystem (MCE) revealed protruding columnar calcareous structures with a *Sinularia* octocoral colony growing atop of each. The current study addressed the hypothesis that these colonies produce spiculites, and sought to determine (a) the spatial occurrence and dimensions of the spiculite-forming colonies and their species affiliation; (b) their microstructural features; and (c) the elemental composition of the columnar spiculites in comparison to the sclerites of the colonies. All the spiculite-forming colonies were exclusively found in the upper MCEs and produced by *S*. *vrijmoethi*. This type of spiculite, including its elemental analysis, is reported here for the first time for coral reefs in general and for the MCE in particular. Examination of the spiculites by scanning electron microscopy and energy-dispersive X-ray spectroscopy revealed spindle shaped-sclerites cemented by crystallites. The elemental composition of the sclerites differed from that of the cementing crystallites, in featuring ~8% Mg in the former and none in the latter. Inductively coupled plasma mass spectrometry revealed fragments of spiculite to be composed of 35% sclerites and 65% crystallites. X-ray powder diffraction analysis of individual sclerites indicated that they are composed exclusively of magnesium-calcite, and the spiculite fragments to also feature 9.3 ± 4% aragonite and 5–7% amorphous calcium carbonate. Consequently, it is proposed that the formation of the crystallites, which lithify the sclerites, is caused by a non-biogenic aragonite precipitation, and that the living colony might benefit from this protruding spiculite structure by means of enhanced exposure to water flow.

## Introduction

Soft corals of the genus *Sinularia* (family Alcyoniidae) are widespread on the Indo-Pacific reefs and considered to be the most speciose among Octocorallia, with more than 185 described species^1^. These species display highly variable growth forms, from encrusting colonies with small knobs or ridges to tall and abundantly lobed ones, occurring in shallow reefs and down to the upper mesophotic coral-reef ecosystems (MCEs)^2,3^. Fleshy octocorals, mainly of the family Alcyoniidae, calcify numerous magnesium (Mg) calcitic sclerites as internal supporting skeletal elements, embedded in their soft coenenchyme^2^. Data on calcium carbonate polymorphs and Mg content in octocorals are sparse. Several Mediterranean gorgonian octocorals feature magnesium calcite as the only calcium carbonate polymorph (1.5–5% Mg, 6–9 mol% MgCO_3_, 0.16–0.025 Mg/Ca)^4^. *Sinularia polydactyla* sclerites were identified in Japan, composed of an Mg-bearing calcite polymorph (5–10 mol% MgCO_3_)^5^. A study on deep-sea octocorals (*Keratoisis* spp., *Lepidisis* spp., and *Paragorgia* spp.) in the south-west Pacific region revealed them to be composed of high-Mg calcite (8–11 mol% MgCO_3_)^6^. The bio-mineralization of octocoral calcitic sclerites in the surrounding high-Mg sea (aragonite sea^7^) was found to be mitigated by specialized extracellular proteins that might prevent Mg^2+^ from inhibiting calcite formation^8^.

Octocorals, therefore, are not usually considered as reef-builders, with a few known exceptions such as *Tubipora musica*, *Heliopora coerulea*, and *Nanipora kamurai*, which all deposit massive aragonite skeletons^2,9^. Some *Sinularia* species, however, have been described as able to consolidate spindle shaped-sclerites at the colony base to form spiculites, which led to the suggestion that certain fleshy octocorals may contribute to reef construction (*e*.*g*. refs^10–12^). A study in Taiwan^13^ recorded 22 spiculite-producing *Sinularia* species. Cores removed from living colonies there revealed a distinct transition in composition from the discrete spindle-shaped sclerites at the top of the colony, to the compact spiculite at the bottom, with its characteristic cementing calcium carbonate. These *Sinularia* octocorals are thus evidently capable of accreting material into significant reef structures over time, and thus can be considered as reef-builders.

In Eilat (Gulf of Aqaba, northern Red Sea), a substantial diversity of *Sinularia* species is common along a wide depth gradient (*e*.*g*. refs^14,15^). During surveys conducted in Eilat’s upper MCEs, live *Sinularia* colonies were found uniquely growing on top of columnar structures, hypothesized here to be spiculites. No shallow colony was found to be growing in a similar fashion. Consequently, the following questions were addressed: (1) What are the spatial occurrence and morphometry of these colonies and their species affiliation? (2) What are the microstructural features of these columnar structures? and (3) What is their elemental composition? The latter two aspects were also examined in comparison with the spindle-shaped sclerites of the studied *Sinularia*. The results shed light on a peculiar feature of soft corals in the MCEs, reflected in the shape of unique calcareous columnar structures, which are suggested to benefit the living colony atop them. These structures have been confirmed to be spiculites, composed of *Sinularia* spindle-shaped calcite sclerites lithified by fibrous aragonite crystallites, whose distinct elemental composition is presented here for the first time.

## Materials and Methods

### Study sites

Surveys were conducted in Eilat at three upper MCE-sites (30–45 m): (a) across from the Interuniversity Institute for Marine Sciences in Eilat (IUI); (b) “Coral Beach” Nature Reserve (NR); and (c) adjacent to the oil jetty of the Eilat Ashkelon Pipeline Company (EAPC) (see Fig. 1 in ref.^3^). The three sites are almost adjacent, separated from one another by 500 m. The IUI site features a moderate slope covered mostly by gravel; the NR- a steep continuous reef wall; and the EAPC- a steep to moderate rocky slope.

**Figure 1:**
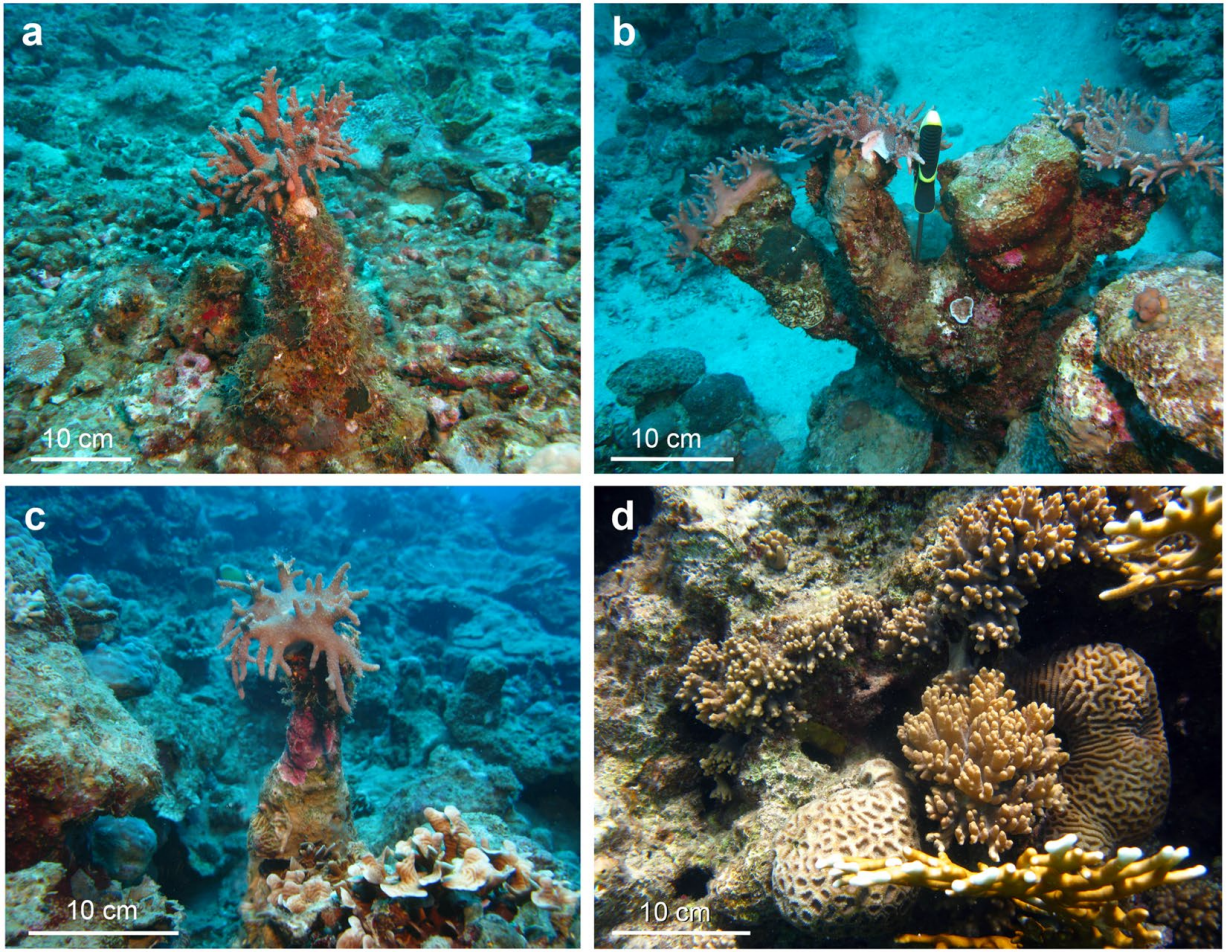
*Sinularia vrijmoethi* in Eilat. (**a**–**c**) Columnar structures bearing a live colony at mesophotic depths (32–44 m). (**d**) Flat and compressed growth form in the shallow reef (3 m).

### Occurrence and morphometry of columnar structures bearing* Sinularia* colonies

To examine the abundance of columnar structures bearing *Sinularia* colonies on their top (Fig. 1a–c), and taking into account that these were found mostly dispersed, 10–30 m apart, the Timed Swims approach was adopted^16,17^, in which two 25-minute dives were conducted at each of the three MCEs. All six dives were carried out at depth of 30–45 m, since no columnar structures had been found in an extensive preliminary survey of the shallower depths (<30 m). Colonies were photographed alongside a measuring tape. Using scaled high-definition photos of the colonies, the height of each columnar structure, from its base on the seabed up to the coral colony base, together with the height and width of the live *Sinularia* colony found on its top (Fig. 1), were measured to the nearest cm. The colony side-view area was then calculated by multiplying its height by the width. The colonies were also sampled for taxonomic identification (see also ref.^3^).

### Microstructure of the columnar structures bearing *Sinularia* colonies and of the spindle shaped-sclerites

Cores, 2 cm in diameter and 5 cm long, were removed from the columnar structures using a pneumatic cup-drill, all perpendicular to their longitudinal axis. Fragments (n = 12, each 3 × 3 × 3 mm) were removed from the cores, immersed in 10% sodium hypochlorite for 10 minutes, followed by repeated rinsing in double-distilled water. The samples were then dried at room temperature. In order to elucidate their microstructural and elemental composition (see below) they were gold-coated and examined by Quanta 200 FEG ESEM (Environmental Scanning Electron Microscope). In addition, small fragments were removed from the respective *Sinularia* colonies found growing on the top of the columnar structures. Spindle-shaped sclerites were removed from these, treated as described above, and examined by ESEM. X-Ray Powder Diffraction was used in order to determine the types of calcium carbonate polymorphs of the cement in the spiculite and in the live colony sclerites. Data were collected using a Bruker d8 advanced difractometer equipped with a LYNXEYE-XE linear detector^18^.

### Elemental analysis

The elemental composition of the spindle-shaped sclerites from both the base of the living colonies and fragments of the columnar structures was examined with an Oxford liquid nitrogen cooled Si(Li) EDS (Energy Dispersive Spectroscopy) detector. Because treating these fragile crystalline features with ordinary polishing methods could obscure certain details, the samples were examined in their native state without polishing. The EDS was performed over crystalline flat facets. The x-ray information volume size due to e-beam interaction (1–10 micron for 20KeV beam energy) was well below the sample size. Element analysis by EDS thus refers to the sample composition. The flat facets were oriented in an optimum sample-detector orientation to derive quantitative analysis for all elements. EDS analysis was carried out while using the conventional EDS standard-less analysis method^19^. This involved using the Oxford company stored data for each element, with the correct element correction factors for the specific sample-detector setup^20^. Evaluation of possible sample destruction/modification by e-beam irradiation was done by secondary electron imaging. The relatively short EDS accumulation time (50–100 sec) provided sufficient EDS signal-to-noise ratio without incurring sample destruction during the measurement.

**Figure 2:**
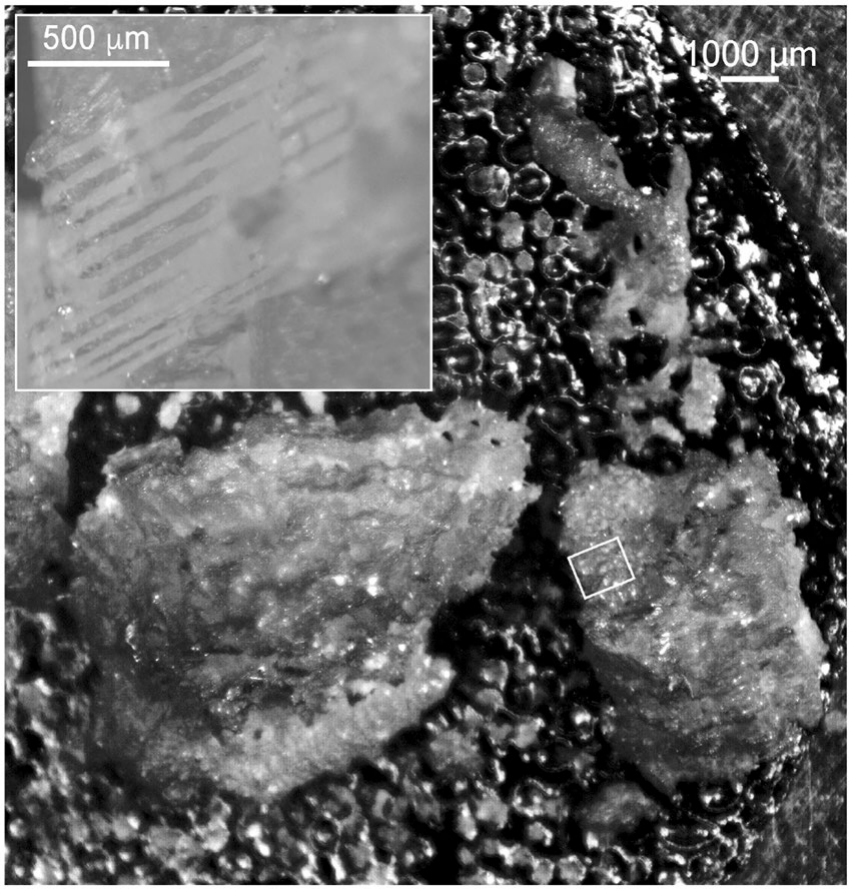
Fragments of a calcareous columnar structure bearing a *Sinularia* colony, mounted on double-sided tape (background) with indication of the area analyzed by laser ablation ICP-MS. Insert showing laser line rasters.

In addition, bulk analysis of spindle-shaped sclerites and fragments of the columnar structures was conducted by digesting 0.1 g of sample using concentrated HNO_3_ (p.a., Merck, Darmstadt, Germany) in a hot plate digestion at 100 °C, and analyzing it by inductively coupled plasma mass spectrometry (ICP-MS) (NexION 350D, PerkinElmer, Waltham, MA, USA). The surface of both types of samples (spindle-shaped sclerites and fragments of the columnar structures) was also analyzed for elemental composition using laser ablation inductively coupled plasma mass spectrometry (LA-ICP-MS)^21^. Before performing the analysis, samples were sonicated for 5 min using reagent grade I water, rinsed with reagent grade I water, air-dried under a laminar hood and stored in pre-cleaned PE vials until analysis. For the analysis, the samples were mounted on microscopic slides using double-sided tape. An ArF excimer laser (New Wave NWR193, ESI, Portland, OR, USA) was coupled to a high resolution ICP-MS (Element XR, Thermo Fisher Scientific, Waltham, MA, USA). Helium was used as carrier gas and mixed with Ar prior to introduction into the plasma torch. Line scans of selected areas of the samples (Fig. 2) were converted into two-dimensional images by using ArcGIS® (version 10.0) following a method recently developed and successfully applied for the advanced spatial analysis of time resolved LA-ICP-MS data (refs^21–24^).

All statistical analyses were performed using R version 3.0.2 and RStudio version 0.98.976. Statistical significance was α < 0.05 for all tests.

## Results

### Occurrence and morphometry of columnar structures

**Figure 3:**
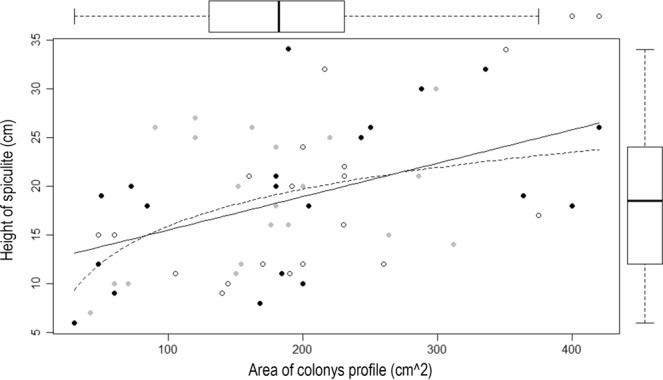
Scatter and box plots of columnar structure height and profile area of their respective *Sinularia vrijmoethi* colonies in Eilat’s mesophotic reefs. Eilat Ashkelon Pipeline Company (gray), Interuniversity Institute for Marine Sciences (blank), and Nature Reserve (black) (n = 22, 19, and 19 structures, respectively). Solid line presents linear model regression with r^2^ = 0.2; and dashed line- logarithmic regression with r^2^ = 0.22 (Pearson correlation, r = 0.45, p < 0.001).

The underwater survey yielded 60 calcareous columnar structures all bearing colonies identified as *S*. *vrijmoethi*: 22 at the EAPC, 19 at the IUI, and 19 at the NR upper MCEs (Fig. 3). Most spiculite-forming colonies were found to be solitary, scattered 10–30 m apart, with only three observations of two-three columnar spiculites having originated next to one another (Fig. 1b). Some spiculites were wider at their basal part due to encrusting calcareous or soft organisms that add an external layer to their outer surface. The colonies featured an average side-view area of 187.7 ± 12.1 cm^2^ (Fig. 3, n = 60, range 42–420 cm^2^). There were no significant differences in colony size among the three sites (Fig. 3, Kruskal-Wallis, p = 0.053). The columnar structures exhibited an average height of 18 ± 0.9 cm (Fig. 3, n = 60, range 5–40 cm) and, similarly, there were no significant differences in height among these MCEs (Fig. 3, ANOVA, p = 0.1). Therefore, the values of all colony side-view area and column-height from the three sites were pooled and a scatterplot and boxplots presenting size frequency distribution were produced (Fig. 3). Analysis revealed a positive and significant correlation between the two parameters (Fig. 3, Pearson correlation, r = 0.45, p < 0.001). Linear regression analysis was used to determine whether the log of the side-view area can significantly predict the respective height of the columnar structure. The results of the regression indicate that the predictor explains 22% of the variance (Fig. 3, R^2^ = 0.22, F(1,58) = 16.02, p < 0.001).

### Microstructure and elemental composition of spindle-shaped sclerites and the columnar structures

**Figure 4:**
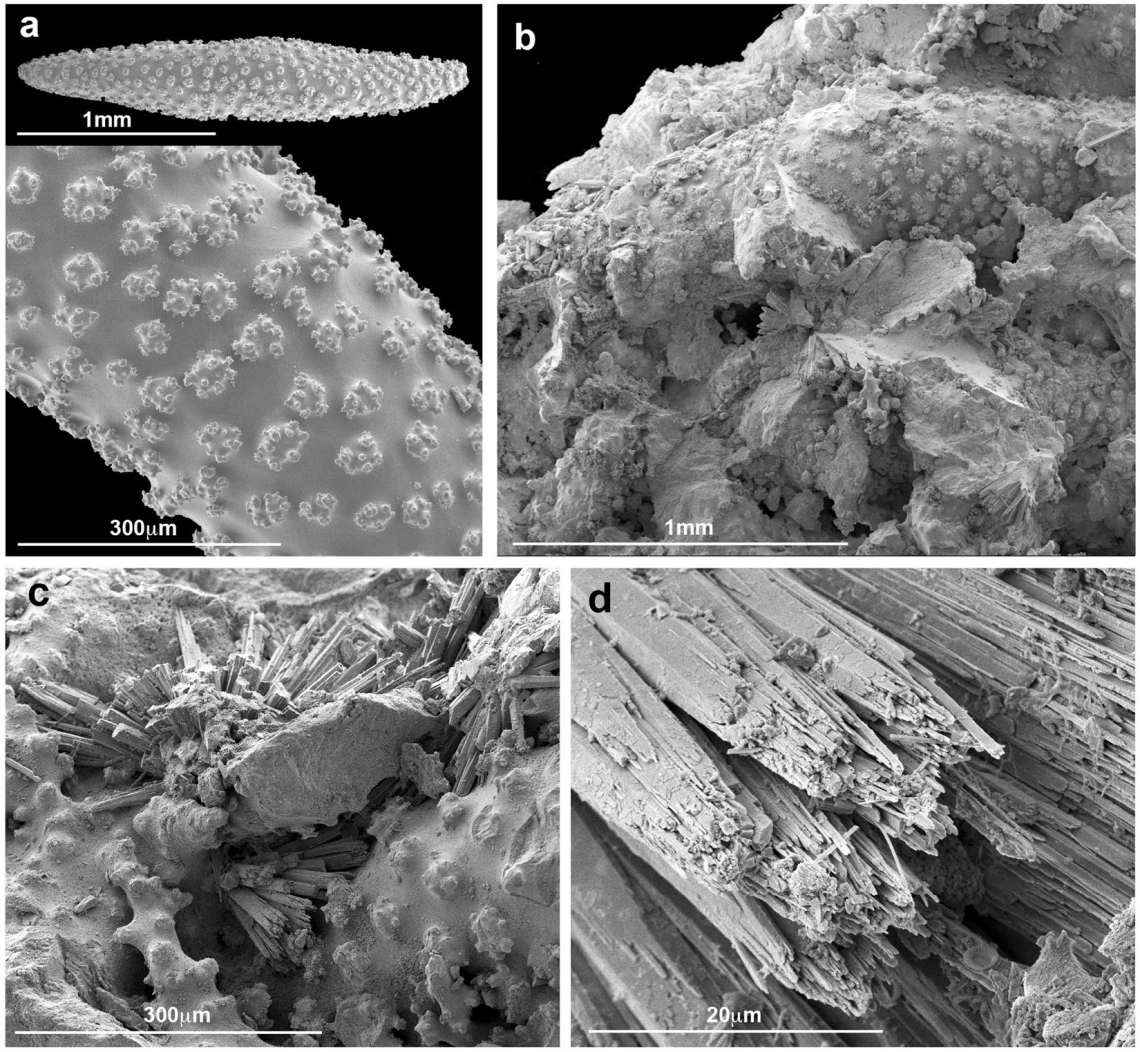
Environmental scanning electron microscope micrographs of fractured spiculite-bearing colony of *Sinularia vrijmoethi*. (**a**) Spindle-shaped sclerite with surface warts. (**b**) Fractured spiculite revealing sclerites lithified by fibrous crystallites. (**c**) Magnified cementing material between spindle-shaped sclerites. (**d**) Fibrous crystallites with euhedral crystal morphology.

ESEM images of the spindle-shaped sclerites from the base of *S*. *vrijmoethi* colonies are presented in Fig. 4a,b. Fragments removed from the columnar structures were found to be constructed of similar spindle-shaped sclerites, cemented by pillar crystals, hereafter referred to as crystallites (Fig. 4b–d). The findings indicate that the columnar structures are spiculites, with the fibrous crystallites lithifying the sclerites into a massive spiculite. The ESEM’s EDS detector analysis revealed that the spindle-shaped sclerites comprising the spiculite contain 7.7 ± 0.3% Mg, whereas those of the crystallites contain only 0.1 ± 0.3% Mg (n = 9 readings each).

The concentration of spindle-shaped sclerites in fragments of the bulk spiculite was obtained by ICP-MS analysis (Table 1). Taking into account the Mg concentration in the spindle-shaped sclerites (77 mg/g), as measured by EDS, and in the crystallites (0.1 mg/g), also in comparison to the spiculite bulk analysis (27 mg/g), it can be concluded that the spiculite fragments are composed of ca. 35% spindle-shaped sclerites and the 65% crystallites.

**Table 1:** Elemental content of spindle-shaped sclerites of *Sinularia vrijmoethi* and of spiculite fragments (bulk analysis using ICP-MS) after digestion; *n* = 3 replicates. Relative measurement uncertainty corresponds to 10% (*U*_rel_; *k* = 2).

Element	Spindle-shaped sclerites	Spiculite fragments	Difference (%)	Unit
Li	1.6	1.6	0	µg/g
Na	1.9	1.9	0	mg/g
Mg	20.5	26.9	31.2	mg/g
Al	1.1	13.9	1163.6	µg/g
K	1.6	0.06	−96.3	mg/g
Ca	156	246	57.7	mg/g
V	0.22	0.15	−31.8	µg/g
Mn	9.5	4.5	−52.6	µg/g
Fe	0.89	1.1	23.6	mg/g
Co	0.22	0.5	127.3	µg/g
Ni	2.2	5.2	136.4	µg/g
Cu	0.33	0.3	−9.1	µg/g
Zn	5.4	2.2	−59.3	µg/g
Rb	0.02	0.1	400	µg/g
Sr	0.96	2.1	118.8	mg/g
Mo	0.17	0.1	−41.2	µg/g
Ba	4.1	8	95.1	µg/g
Tl	0.16	0.02	−87.5	µg/g
Pb	0.04	0.29	625	µg/g
U	0.04	0.32	700	µg/g

**Figure 5:**
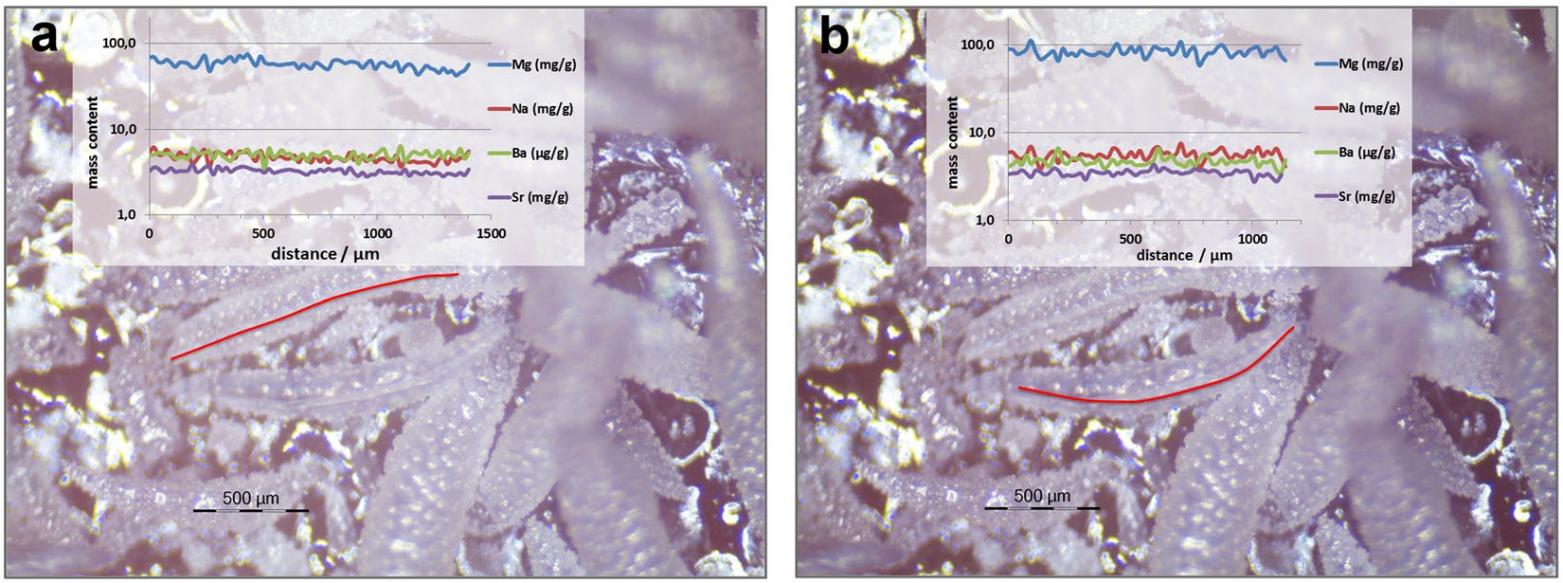
Laser ablation ICP-MS profiles with concentrations of Na, Sr, Ba, and Mg along two spindle-shaped sclerites isolated from an *Sinularia* *vrijmoethi* colony tissue.

Laser ablation ICP-MS profile of a single spindle-shaped sclerite of *S*. *vrijmoethi* (Fig. 5) revealed a highly homogeneous distribution of the following measured elements: Na, Mg, Sr, and Ba in the Ca matrix (average relative standard deviation of 10%). The average elemental content of Na, Sr, Ba, and Mg as determined by LA-ICP-MS is presented in Table 2.

**Table 2:** Elemental content of Na, Mg, Sr, and Ba in two spindle-shaped sclerites of *Sinularia vrijmoethi* obtained by Laser ablation ICP-MS.

Element		Na	Mg	Sr	Ba
Sclerite 1	(mg/g)	4.6	56.53	3.17	5.00
	RSD	10%	13%	7%	11%
Sclerite 2	(mg/g)	5.6	82.88	3.49	4.69
	RSD	13%	12%	8%	12%

**Figure 6:**
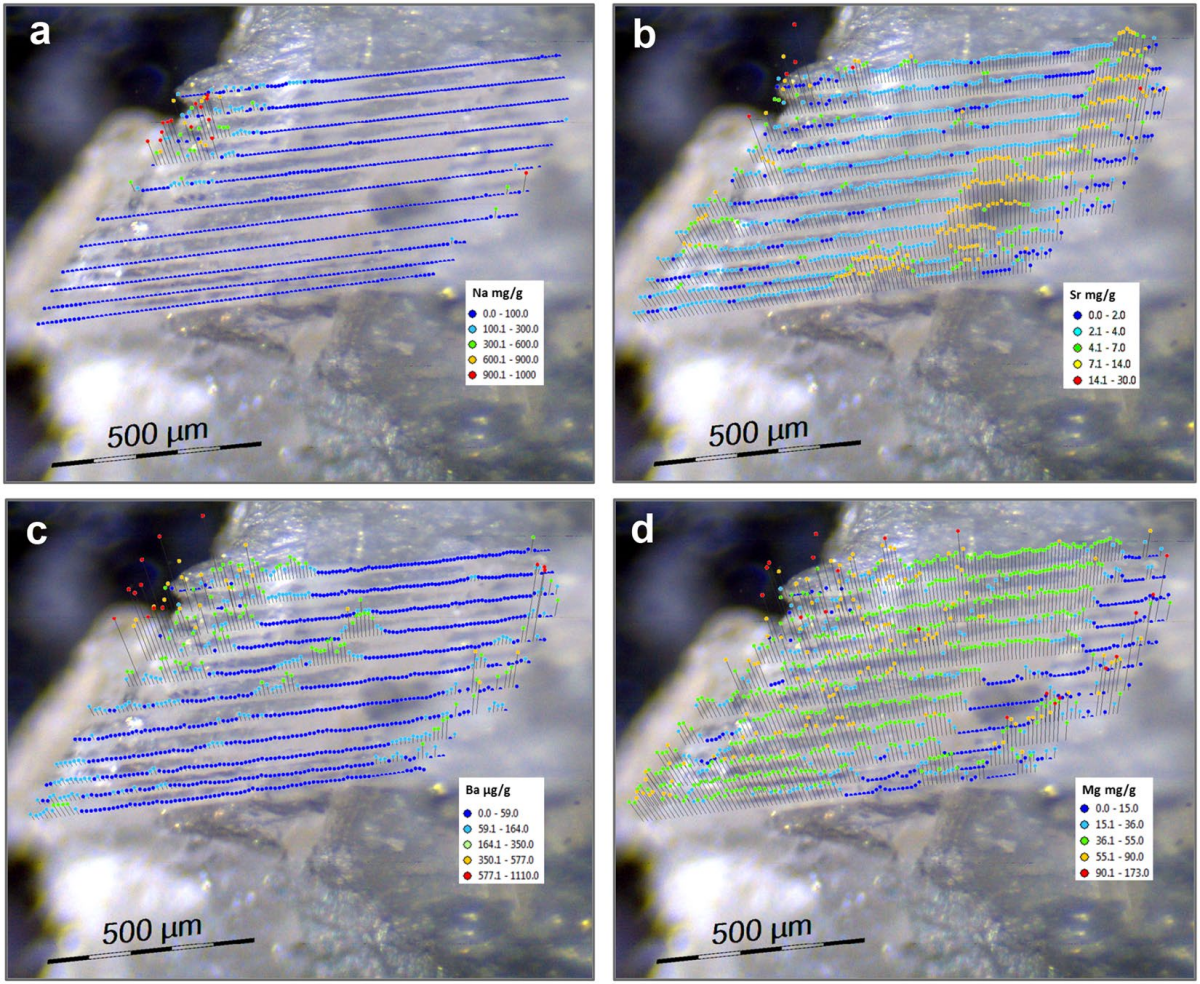
Laser ablation ICP-MS lateral distribution of (**a**) Na, (**b**) Sr, (**c**) Ba, and (**d**) Mg on selected spiculite area.

The lateral distribution of Na, Sr, Ba, and Mg on the selected spiculite area is presented in Fig. 6.

### XRD analysis

**Figure 7:**
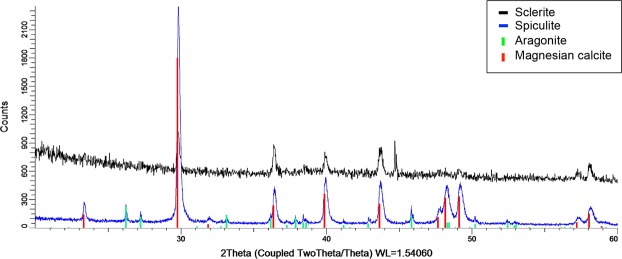
X-ray powder difraction analysis of sclerites from the living colony (black) and of spiculite fragments (blue). Reference markings are shown for aragonite (green, PDF 00-041-1475) and magnesium-calcite (red, PDF 01-086-2336).

Two samples were analyzed: sclerites isolated from the living colony and spiculite fragments taken from the middle part of the column. In both samples, magnesium-calcite was identified (Fig. 7) while aragonite was found only in the spiculite fragments. Using Rietveld refinement calculation, 9.3 ± 4% of the spiculite fragments was found be aragonite, and from the background interpolation, the presence of about 5–7% amorphous calcium carbonate is estimated. The structural parameters determinate for the aragonite phase are: grain size = 101(12) nm; a = 4.9671(9) Å; b = 7.977(2) Å; c = 5.7538(8) Å. For the magnesium-calcite phase: grain size = 53.4(7) nm; a = 4.9348(4) Å; c = 16.822(2)Å.

## Discussion

The findings of the current study confirmed the hypothesis that colonies of *S*. *vrijmoethi* produce columnar spiculites in Eilat’s upper MCEs. Although spiculites have been reported in previous studies, this particular type of columnar spiculite is a feature uniquely found at mesophotic depths, and is reported here for the first time. In his study, Konishi^10^ did not provide the species affiliation of the spiculite-producing octocoral he reported, nor any morphometric details; Schuhmacher^11^ reported that *S*. *leptoclados* and *S*. *minima* form spiculites in the Sudanese Red Sea, found from shallow water down to 35 m. These were up to 110 cm long and 20 cm wide, but differ morphologically from those described in the present study; Cornish & DiDonato^12^ studied the sclerite masses noted by Cary^25^ and referred them to spiculites of *S*. *polydactyla* found in shallow reefs, but they did not record their abundance or morphometry; and Jeng *et al*.^13^, who reported spiculites at depths of 10–15 m on southern Taiwan reefs, concluded that the calcification process leads to cementation of the sclerites, which takes place at the base of the colony and differs from sclerite formation. In all the above studies, *Sinularia* spiculites were reported to form large masses, with none resembling the columnar ones produced by S. *vrijmoethi* found in Eilat’s upper MCEs. The lack of such spiculite in Eilat’s well-studied shallow reefs suggests that it may serve as an adaptation of this species in the upper MCEs (see below).

The current study recorded a range of spiculite heights in Eilat’s upper MCE (Fig. 3: 18 ± 0.9 cm), albeit smaller than those reported in previous studies: ^10^: 110 cm, ^11^: “continuous reefs”, ^12^: “large boulders”. The noticeably small colony-size and spiculite dimensions in Eilat might derive from a low calcification rate in the upper MCEs, a subject that awaits future studies. The colony size of *S*. *vrijmoethi* corresponded to the respective spiculite height (Fig. 3), suggesting a direct and non-linear relationship between the two. Nonetheless, only 22% of the variation in spiculite height is explained by colony size, probably due to the distinct morphology of *S*. *vrijmoethi* colonies in the MCE. Their morphology differs from that of the shallow-reef colonies, where they form beds composed of aggregated colonies of various sizes (Fig. 1d). It appears that the MCE colonies may suffer from physiological constraints (i.e. light^26^) that might limit their horizontal growth, as opposed to the shallow reef. However, they grow on the top of the spiculite, featuring a nearly round morphology and continuously depositing sclerites, thus lengthening the spiculite underneath. This, in turn, might benefit the colonies through exposure to a better water flow compared to that prevailing on the seabed and consequently to an increased nutrient and plankton flow^27^. The advantages of this increase in elevation and the possible shift of octocoral nutrition from autotrophic in the shallow reef to heterotrophic in the MCEs, are the subject of our ongoing research. The disadvantages might include (in cases in which the spiculite cementation rate is lower than the colony growth rate) a reduction in available substrate for the colony base, ultimately limiting colony size.

Some spiculites are wider at their base, being encrusted by organisms such as calcareous algae, bryozoans, and sponges. At the steep NR and EPCA MCE sites the spiculites are found among the stony-coral colonies and therefore contribute to the complex reef structure. In the IUI MCE plateau, which is covered with gravel, the vertical spiculites emerging from the flat bottom are distinct due to their vertical nature.

As most of the differences between sites can be attributed to substrate and reef steepness, as described in the Methods section, the Results indicate that the abundance and size of the *Sinularia* colonies that form spiculites is unaffected by these factors. As opposed to the shallow upper fore-reef in Eilat, where *S*. *vrijmoethi* colonies are common, grow in patches and do not consolidate columnar spiculites^3^, in the MCE, spiculite–forming colonies were found to be solitary and individually scattered 10–30 m apart, with three exceptions of spiculites having originated next to one another (Fig. 1b).

Similarly to Jeng *et al*.^13^, the current study presents images of fractured fragments removed from the spiculites (Fig. 3). Octocoral sclerites are composed of magnesium-enriched calcite, with an Mg content of 12–15%^5^; and are known to possess a striking micrometric-scale concentration-zoning of this element^28^. In the current study, the elemental composition of *S*. *vrijmoethi* sclerites differed from that of the crytallites, with the former containing ~8% Mg, as opposed to almost none in the latter. Consequently, it is suggested that the formation of the fibrous crystallites is a purely chemical action, essentially being an inorganic precipitation of aragonite^29^.

The combination of data obtained by ICP-MS (bulk elemental analysis) with the EDS analysis enabled us to determine the composition of the studied samples as comprising 35% sclerites and 65% crystallites. Analysis of the spindle-shaped sclerites revealed their homogenous elemental distribution as well as their homogeneous composition per sclerite. The elemental content in the different spindle-shaped sclerites varied within 10% for Na, Sr, and Ba, while the variation in Mg was higher (>30%).

The lateral distribution within the spiculite surface area revealed a regular distribution of Na. However, that of Sr, Ba, and Mg featured small areas with increased elemental content, supporting the hypothesis of the presence of small crystalline Mg-rich regions (see Fig. 4b–d). The overall conclusion is that the spindle-shaped calcite sclerites are cemented by euhedral crystallites featuring fibrous aragonite. A similar cement was recorded by Grammer *et al*.^30^ as exhibiting rapid rates of cementation with far-reaching implications for the morphology of some carbonate substrates in the marine environment. Undoubtedly, the dynamics of the lithification process of sclerites requires future studies, also in relation to the prevailing physical and chemical conditions in the MCEs.

The position of *S*. *vrijmoethi* colonies on top of the columnar spiculite is suggested to correspond to the prevailing flow regime, in which maximum flow prevails above the seabed of the upper MCE. The spiculite morphometry may thus benefit the colony on its top, providing it with access to a suitable flow regime for the effective capture of planktonic food. As spiculites have not been recorded in the shallow reefs of Eilat, further studies on this topic will undoubtedly shed light on the adaptive significance of the spiculite morphometry to the environmental conditions of the upper MCEs. Consequently, it is argued that the significance of spiculites in the reef environment is beyond that of merely their hermatypic function.

## Data Availability

The datasets generated and/or analyzed during the current study are available from the corresponding author upon request.
